# ANCA-associated vasculitis following Oxford-AstraZeneca COVID-19 vaccine in Brazil: Is there a causal relationship? A case report

**DOI:** 10.3389/fmed.2022.1003332

**Published:** 2022-10-06

**Authors:** Welder Zamoner, Julia Baldon Scardini, Bruna Jordana De Dio, Amanda de Melo Marques, Vanessa dos Santos Silva, Aline Lutz Garcia, Daniela Cristina dos Santos, Rosa Marlene Viero

**Affiliations:** ^1^Department of Internal Medicine, Discipline of Nephrology, Botucatu School of Medicine, University São Paulo State—UNESP, Botucatu, São Paulo, Brazil; ^2^Department of Pathology, Botucatu School of Medicine, University São Paulo State—UNESP, Botucatu, São Paulo, Brazil

**Keywords:** acute kidney injury, COVID-19, vaccine, Oxford, AstraZeneca

## Abstract

This article presents a case of rapidly progressive glomerulonephritis following the Oxford-AstraZeneca COVID-19 vaccine in a female patient 58 years old. After 5 days, she presented fatigue, paleness, arthralgia on hands, knees, ankles, foamy urine, and elevated blood pressure. Exams showed serum creatinine of 2.2 mg/dL (baseline creatinine of 1.0 mg/dL). Urinalysis revealed hematuria, and her 24-h urinary protein excretion was 4.4 g. Additional exams showed hypercholesterolemia, severe anemia, and normal serum albumin. Testing of antineutrophil cytoplasmic antibodies anti-myeloperoxidase was positive at a titer of 1/80. Serum and urine protein electrophoresis and other exams showed no alterations. She was started on steroid pulse therapy after worsening kidney function, reaching serum creatinine of 3.3 mg/dL. A kidney biopsy revealed crescentic glomerulonephritis with glomerular sclerosis, fibrous crescents, interstitial fibrosis, and tubular atrophy. Induction therapy was given with intravenous cyclophosphamide 0.5 g/m^2^ for 6-monthly pulses, followed by maintenance therapy with oral azathioprine at 2 mg/kg and prednisone tapering. The patient did not develop any complications during the induction therapy, and is currently on maintenance therapy with a serum creatinine of 1.87 mg/dL.

## Introduction

Coronavirus (SARS-CoV-2) infection, named COVID-19, was detected for the first time in China in December 2019 and has affected over 380 million people worldwide, causing over 5.7 million deaths. In Brazil, over 25.7 million cases and 628 thousand deaths have been reported, primarily from Severe Acute Respiratory Syndrome.

The pandemic caused a significant social impact, and the search for immunization became fundamental.

Although acute kidney injury related to COVID-19 infection is frequent, its association with vaccines is rare. This article presents a case of rapidly progressive glomerulonephritis following vaccination.

## Case report

The patient was female, 58 years old, with a previous medical history of hyperthyroidism treated in 2006, and at the moment of evaluation, not on any medication. She received the first dose of Oxford-AstraZeneca Covid vaccine and developed a minor reaction (myalgia and pain on the injection site) in the following 2 days. After 5 days, she presented fatigue, paleness, arthralgia on hands, knees, ankles, foamy urine, and elevated blood pressure.

Due to persisting systemic symptoms, she sought medical attention. Investigation revealed a serum creatinine of 2.2 mg/dL, urea of 67 mg/dL, a significant elevation compared to a baseline creatinine of 1.0 mg/dL. Urinalysis revealed hematuria (20 to 25 red blood cells per high power field) and proteinuria (2+). The 24-h urinary protein excretion was 4.4 g. Additional investigations showed hypercholesterolemia, severe anemia, and normal serum albumin. There was a hematological investigation, with negative hemolysis tests, marrow aspirate and immunohistochemistry demonstrating reactive marrow, absence of myelodysplasia or neoplasia in the sample. Because of the evidence of altered kidney function, she was referred to a Nephrologist.

Ultrasound showed kidneys with 10.5 cm long and parenchyma 1.6 cm size. Antinuclear antibody test (ANA) and anti-double-stranded DNA (anti-dsDNA) were negative. The serum complement (C3 and C4) were within normal limits, and testing of antineutrophil cytoplasmic antibodies (ANCA) anti-proteinase 3, anti-glomerular basement membrane (GBM) and viral serologies were negative. However, anti-myeloperoxidase was positive at a titer of 1/80. Serum and urine protein electrophoresis showed no alterations.

She was started on steroid pulse therapy after worsening kidney function, reaching serum creatinine of 3.3 mg/dL and indicating possible rapidly progressive glomerulonephritis (RPGN). She received 1 gram of methylprednisolone daily for three consecutive days in an outpatient setting, followed by 1 mg/kg of oral prednisone. She required a blood transfusion, vitamin B12, folic acid supplementation, and medication to reduce blood pressure during follow-up.

**Figure 1:**
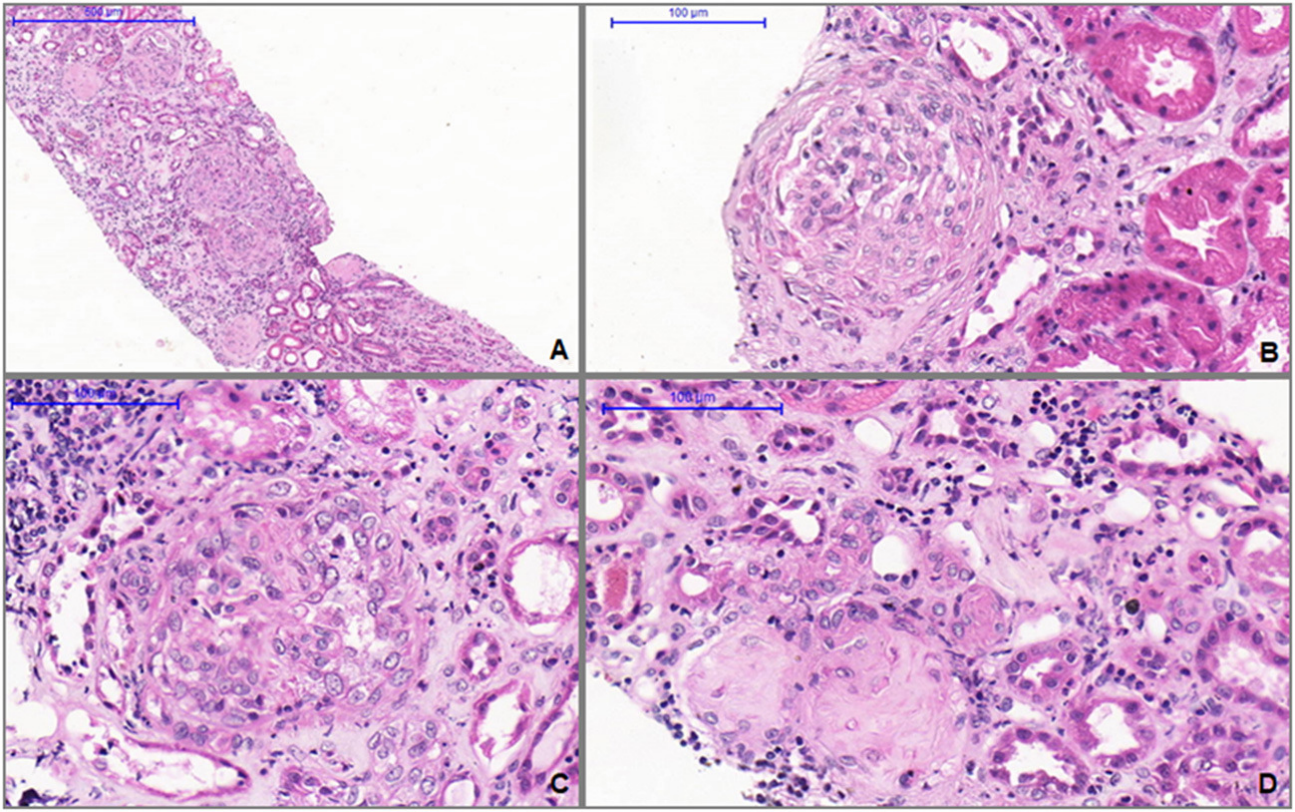
**(A)** Light microscopy shows glomerular sclerosis, fibrous crescents, interstitial fibrosis, and tubular atrophy (H&E, 100X). **(B)** Glomerulus with fibrocellular crescents (H&E, 400X). **(C)** Active glomerular lesion showing endo and extracapillary hypercellularity (H&E, 400X). **(D)** Thickened and tortuous arteriole associated with globally sclerotic glomerulus and extensive parenchymal atrophy and fibrosis (H&E, 400X).

A kidney biopsy was performed 15 days after initiation of treatment and 80 days after vaccine administration (Figure 1) and revealed crescentic glomerulonephritis with glomerular sclerosis, fibrous crescents, interstitial fibrosis, and tubular atrophy (Figures 1A,B). We also observed in the glomeruli active inflammatory lesions characterized by endothelial swelling, endocapillary proliferation, accumulation of macrophages, hyaline deposits and cellular e fibrocellular crescents (Figures 1B–D). Immunofluorescence confirmed nonspecific entrapment with C3 positive in sclerotic areas.

After the biopsy, induction therapy wasgiven with intravenous cyclophosphamide 0.5 g/m^2^ for six-monthly pulses followed by maintenance therapy with oral azathioprine at 2 mg/kg and prednisone tapering.

The patient did not present any complications during the induction therapy, and outpatient follow-up continues maintenance therapy with a current serum creatinine of 1.87 mg/dL.and with 24-h urinary protein excretion of 0.5 g.

## Discussion

As vaccination advances, some side effects have been reported (1–3). Most common among them are tenderness on the injection site, fever, fatigue, myalgia, and headaches (2). The association between COVID-19 and kidney impairment has been well established.

Some vaccines have been associated with the development of autoimmune diseases following immunization, including reports of ANCA-associated vasculitis (AAV) after Influenza vaccination (1, 3). AAV is characterized by small-vessel vasculitis and the presence of antineutrophil cytoplasmic antibodies (2). To this date, episodes of AAV following the vaccines from Pfizer (1) and Moderna (4) have been reported.

The mechanism behind this association is uncertain. It could be explained by molecular mimicry, polyclonal activation of B cells, or transient proinflammatory cytokines response, leading to autoimmune diseases in genetically predisposed individuals (2, 5).

The biopsy identified changes in appearance in chronification, compatible with the degree of aggressiveness of lesions of increasing vasculitis with significant impairment of renal function. The unclear etiology of anemia was also identified with the possible contribution of systemic inflammatory reaction.

Here we presented a case of AAV following immunization against SARS-CoV-2 with the Oxford-AstraZeneca vaccine. Although this was a temporally related fact in a patient with previously normal renal function, suggesting de novo vasculitis, it is impossible to rule out previous renal alterations due to vasculitis or other undiagnosed issues. Causality is based solely on temporal precedence, as a direct correlation to the vaccine cannot be proved.

## Data availability statement

The raw data supporting the conclusions of this article will be made available by the authors, without undue reservation.

## Ethics statement

The studies involving human participants were reviewed and approved by Comitê de Ética em Pesquisa - Faculdade de Medicina de Botucatu. The patients/participants provided their written informed consent to participate in this study.

## Author contributions

WZ, JS, and BD contributed to conception and design of the study. AM and VS organized the database. WZ, JS, BD, and AM wrote the first draft of the manuscript. AG, DS, and RV wrote sections of the manuscript. All authors contributed to manuscript revision, read, and approved the submitted version.

## Conflict of interest

The authors declare that the research was conducted in the absence of any commercial or financial relationships that could be construed as a potential conflict of interest.

## Publisher's note

All claims expressed in this article are solely those of the authors and do not necessarily represent those of their affiliated organizations, or those of the publisher, the editors and the reviewers. Any product that may be evaluated in this article, or claim that may be made by its manufacturer, is not guaranteed or endorsed by the publisher.
